# Taxes for tuberculosis: could tobacco and sugar tax revenue fund tuberculosis control interventions?

**DOI:** 10.1136/bmjgh-2025-019770

**Published:** 2025-09-08

**Authors:** Mikaela Coleman, Anna K Coussens, Claire Jacqueline Calderwood, Ingrid Schoeman, Madhavi Bhargava, Pranay Sinha, Ben J Marais, Katharina Kranzer

**Affiliations:** 1 Institute of Infectious Diseases and Tropical Medicine, LMU University Hospital, LMU Munich, Munich, Germany; 2 Sydney Infectious Diseases Institute (SydneyID), The University of Sydney, Sydney, New South Wales, Australia; 3 Infection and Global Health Division, Walter and Eliza Hall Institute of Medical Research (WEHI), Department of Medical Biology, University of Melbourne, Parkville, Victoria, Australia; 4 Department of Medical Biology, University of Melbourne, Parkville, Victoria, Australia; 5 Wellcome Research Discovery Platforms in Infection, Institute of Infectious Disease and Molecular Medicine, Department of Pathology, Faculty of Health Sciences, University of Cape Town, Cape Town, South Africa; 6 Clinical Research Department, London School of Hygiene & Tropical Medicine, London, UK; 7 The Health Research Unit Zimbabwe, Biomedical Research and Training Institute, Harare, Zimbabwe; 8 TB Proof, Pretoria, South Africa; 9 Department of Community Medicine, Yenepoya Medical College, Yenepoya (Deemed to be University), Yenepoya, India; 10 Boston Medical Center, Boston, Massachusetts, USA; 11 Boston University Chobanian & Avedisian School of Medicine, Boston, Massachusetts, USA; 12 WHO Collaborating Centre for Tuberculosis, Sydney, New South Wales, Australia

**Keywords:** Tuberculosis, Tobacco, Nutrition, Diabetes, Health policy

**SUMMARY BOX**

- Elimination of tuberculosis requires significant long-term funding.
- International sources of funding are increasingly precarious for countries with high-tuberculosis incidence.
- Tobacco and sugar taxes generate domestic revenue and simultaneously reduce tuberculosis vulnerability (eg, lung damage, diabetes, undernutrition).
- With advocacy, revenue from tobacco and sugar taxes could fund sustainable community-wide tuberculosis and nutrition interventions.

## Introduction

 Tuberculosis (TB) is the number one infectious killer on the planet while post-TB lung disease affects the quality of life and work capacity of millions every year.^1^ Active and passive tobacco smoking increases the risk of TB and subsequent long-term pulmonary sequelae.^2^ Undernutrition is the leading driver of the TB pandemic globally,^1^ predisposing those with *Mycobacterium tuberculosis* infection to the development of TB and those with TB to worse outcomes. Moreover, diabetes, driven by high-sugar, ultra-processed diets, is another leading determinant of TB, accelerating in prevalence worldwide.^3^

This syndemic (‘simultaneous epidemic’) of TB and malnutrition is expensive for patients and governments. Catastrophic costs associated with TB and post-TB disability exacerbate poverty among affected individuals and families.^4^ Global economic losses from TB mortality will amount to trillions of dollars if TB elimination remains ‘off track’ in coming decades.^5^ Population-wide TB screening and nutrition interventions are promising^6 7^ but are associated with huge ‘start-up’ costs—even if cost saving in the long run. Critically, given that TB elimination will require a reduction in community vulnerability (including tobacco use) and reliable long-term funding to support active case finding and treatment, it is important to consider interventions that may achieve both.

## The need for sustainable funding

The 2025 funding cuts in the USA aid funding and similar actions in the UK and elsewhere highlight the fragility of relying on donor support for chronic and endemic diseases like TB.^8 9^ Even well-established external funding can be abruptly disrupted, leaving high-burden countries vulnerable. While the Global Fund has strongly supported TB screening, prevention and care, strategies that ensure long-term sustainability and prevent donor funds from merely replacing existing resources remain a challenge. Many countries continue to juggle fragmented, short-term TB project funding from multiple donors, leading to duplication and inefficiencies. Scalable, locally tailored TB and nutrition interventions with high coverage require sustained, substantial investment—funding that remains largely unavailable in most high TB burden regions.

This poses the question: is there a regenerative domestic funding source that can be dedicated to combat the TB-malnutrition syndemic? This funding may already exist in many settings worldwide in the form of tobacco and sugar taxes, but the dedication of this tax revenue to benefit population health is an opportunity that remains underexplored.

## Taxing ‘Big Tobacco’ and ‘Big Sugar’

Tobacco and sugar/ultra-processed food have catastrophic impacts on TB incidence and mortality through impaired lung health, malnutrition and diabetes. Multinational tobacco and sugar industries invest substantial resources into creating environments that normalise harmful consumption, wielding advertising, political lobbying, manipulative industry-funded research, competitive pricing, repackaging and reformulations that maximise addictiveness.^10^ Their targeting of people living in low-income and middle-income countries is well-documented—settings that also bear the greatest TB burden globally.^1 10^ These industries must be held financially accountable for remediating the harm they have inflicted on communities.

Over a 10-year time horizon, tobacco taxes have more than halved tobacco use in various global settings, reducing vulnerability to TB and other smoking-related diseases and diminishing under-5 deaths in countries from all wealth quintiles.^11 12^ Importantly, a price increase of only one international dollar per cigarette pack across 181 countries could increase global health expenditure by 4% if this revenue were dedicated to local health budgets.^13^ A study in just four states in India estimated that higher cigarette excise taxes could generate an additional US$600 million in revenue—double the TB funding for the entire country in 2023.^14^ The earmarking of tobacco and alcohol tax revenue for healthcare insurance in the Philippines under the Sin Tax Reform Law 2012 provides an important proof of concept that could be expanded to support TB and nutrition interventions globally. Likewise, more recently introduced sugar taxes have proven effective in reducing sugar consumption, with anticipated impacts on type 2 diabetes and obesity.^15^ In South Africa, the purchase of sugary beverages dropped by 57% in urban socioeconomically deprived households following a 10% sugar tax.^16^ Tobacco and sugar tax revenue should be reinvested into the health of the communities exploited by these industries.

Low-middle-income countries also stand to make the greatest gains from a tobacco or sugary beverage tax. Estimates suggest a 50% increase in real product prices could raise overall government healthcare spending by 40% if revenue is appropriately earmarked for health in these settings.^17^ As taxes diminish harmful consumption over time, so too does related health expenditure. Although this may shrink the tax base over time, this would be a good problem to have. Most high TB incidence settings have no or very low existing tobacco and sugar taxes with huge scope to generate tax revenue without harmful consequence. The WHO recommendation suggests 75% taxation, which demonstrates the scope for revenue generation and longer-term sustainability.^18 19^ Despite the complexities of efficient implementation and appropriate earmarking, the global TB community must seriously consider this opportunity, given the urgent funding need and well-established proof-of-principle.^20^

## Funding TB syndemics

Tobacco and sugar taxes, if implemented carefully and equitably (table 1),^21^ can curb harmful consumption—especially among vulnerable populations—while generating sustainable funding for lung health and nutrition programmes. Redirecting this revenue towards TB screening, food subsidies and other holistic prevention-oriented interventions can help shift from a ‘scarcity mindset’—where limited resources are hoarded for vertical TB biomedical interventions—towards a syndemic approach.^22^ The syndemic approach considers the reality of TB as a condition of overlapping vulnerabilities, offering high-incidence countries a transformative path toward TB elimination. This combination of anti-TB and pro-nutrition activities, together with sustained domestic financing, presents a golden opportunity for high-incidence countries to take decisive action to bring TB elimination within reach.

**Table 1:** Risks and mitigation strategies to consider when raising taxes to support disease control initiatives

Sugar/ultra-processed food taxes Lack of availability of alternatives to ultra-processed or sugar-sweetened foods in communities, leading to dependence on these foods for caloric intake. Price increases consequent on introduced taxation have disproportionate impact on the poorest, with poorer communities unable to afford food.	Prioritise research to find evidence-based strategies for taxation introduction that minimise inequalities and are tailored to population and food system contexts, guided by existing resources such as the WHO Manual on Sugar-sweetened Beverage Taxation Policies to Promote Healthy Diets 2022. Use tax revenue to fund school food programmes, nutritious food subsidies and partnerships with departments of Agriculture and Trade to support local sustainable and micronutrient-rich farming (eg, pulses) and drive down costs for households. Use the WHO operational guidance on the multisectoral accountability framework to end TB 2023 and other multisectoral engagement tools to maximise government buy-in and community health impact of taxes. Extend taxation to limit industry advertising, ‘sportswashing’ sponsorships and to sanction industry-affiliated partners, preventing industry from ‘handing over’ entire cost of the tax to consumers.
Tobacco taxation Price increases make smoking more costly, with the greatest impact on vulnerable groups (eg, people who are poor or mental health conditions) who have other strong social and structural drivers for smoking, and the highest barriers to quitting. Public messaging around taxation may increase stigma and discrimination of people who smoke. Tax levy is not sufficiently high to disincentivise consumption. Negative effect on wages and employment opportunities in regions economically dependent on the tobacco supply chain.	Use tobacco tax revenue to fund equitable, holistic smoking cessation support, with a focus on meeting the needs of the most vulnerable and reaching particularly affected areas and groups. Tobacco industry itself targets disadvantaged people; the bigger economic burden in this group correlates to a greater incentive to quit among the vulnerable population—invest in branding/messaging that emphasises industry responsibility for smoking. Follow current evidence for greatest success using the WHO Framework Convention on Tobacco Control, aiming to tax at least 75% of the retail cost of these products.^23^ Pre-empt common industry tactics, working simultaneously with employment to prevent wage reductions within tax-sanctioned industries.

TB, tuberculosis.

## Conclusions

Implementation of tobacco and sugar taxes requires collaboration and investment by many political actors as well as policies that promote transparency and accountability in the use of this tax revenue. The TB community should advocate for the implementation of such taxes in TB high-incidence settings and lobby for resultant revenue to be invested in public health interventions which support TB elimination. Country context-tailored taxes supported by robust monitoring and accountability frameworks could provide the regenerative source of funding needed to bring TB elimination within grasp. ‘Taxes for tuberculosis’ represents an innovative and bold funding opportunity that prioritises what is possible, durable and local.

## Data Availability

Data sharing is not applicable to this article as no new data were created or analyzed in this commentary.
